# Risk-directed therapy based on genetics and MRD improves the outcomes of AML1-ETO-positive AML patients, a multi-center prospective cohort study

**DOI:** 10.1038/s41408-023-00941-4

**Published:** 2023-11-13

**Authors:** Dan Xu, Ying Yang, Zhao Yin, Sanfang Tu, Danian Nie, Yiqing Li, Zhenqian Huang, Qixin Sun, Changfen Huang, Xiaqi Nie, Zurong Yao, Pengcheng Shi, Yu Zhang, Xuejie Jiang, Qifa Liu, Guopan Yu

**Affiliations:** 1 Department of Hematology, Nanfang Hospital, Southern Medical University, Guangzhou, China; 2 Department of Hematology, Zhujiang Hospital, Southern Medical University, Guangzhou, China; 3 Department of Hematology, Sun Yat-Sen Memorial Hospital, Sun Yat-Sen University, Guangzhou, China; 4 Department of Hematology, The First Affiliated Hospital of Guangzhou Medical University, Guangzhou, China; 5 Department of Hematology, Guangzhou First People’s Hospital, Guangzhou, China

**Keywords:** Leukaemia, Medical research

**Dear Editor**,

Acute myeloid leukemia (AML) with AML1/ETO-positive or translocation (8;21) (AE-AML) has been shown clinical heterogeneity [1–4] and is necessary to be further stratified. Numerous studies [3, 5–7] reveal the importance of measurable residual disease (MRD) as a prognostic predictor in guiding tailor treatment of AE-AML, that patients with high MRD levels are categorized as high-risk and receive allogeneic stem cell transplantation (allo-SCT), while those with low MRD are low-risk and receive chemotherapy (CT), and finally significantly improve the long-term survival of the whole cohort [8, 9]. Combination of genetics and dynamic MRD levels to guide risk stratification is well accepted in the treatment of AML [8, 10–13]. Whether it also works in AE-AML remains unsure. Most recently, one study combined KIT mutation with MRD in risk stratification and improved treatment guidance in AE-AML [12]. As known, KIT mutation is a widely accepted molecular event with strong prognostic significance in AE-AML [14–18]. Besides, Krauth, M.T. et al. reported that ASXL1 mutation was another independently adverse factor for AE-AML [4]. In accordance, our previous study [2] also found that KIT and ASXL1 mutations poorly influenced the prognosis in patients with AE-AML, especially poorer in those with co-mutation of KIT and ASXL1, in agreement with the synergism of KIT and ASXL1 mutations in the development of AML driven by the AML1-ETO fusion gene [19, 20]. Thus we conducted a prospective multi-center cohort study to investigate whether risk-directed treatment according to KIT/ASXL1 mutations and MRD levels could decrease relapse and improve survival of AE-AML patients, and then provide a stratification treatment strategy for improving the prognosis.

Study design was as follows, patients were first divided into KIT−ASXL1− (non-mutation), KIT+/ASXL1+ (single-mutation), and KIT+ASXL1+ (co-mutation) groups according to mutation detection with the next generation sequencing (NGS) as our previous reports [11, 21]. After acquiring complete remission (CR) or CR with incomplete blood count recovery (CRi), patients were recommended to receive risk-directed therapy based on KIT/ASXL1 mutations and MRD levels after two cycles of consolidation with high dose cytarabine [16, 22]. Main molecular response (MMR) was defined as >3 logs reduction of MRD levels as compared with the pre-treatment and the AML1-ETO transcript levels <0.1%, detected with quantitative polymerase chain reaction (qPCR) [2, 11]. Low-risk (LR) patients (KIT−ASXL1− with MMR) received CT or autologous (auto-) SCT, intermediate-risk (IR) patients (KIT+/ASXL1+ with MMR) took auto-SCT or human leukocyte antigen (HLA) matched allo-SCT, high-risk (HR) patients (KIT+ASXL1+ or without MMR) were bridged to allo-SCT including HLA-matched or haploidentical (haplo−). The protocol was approved by the ethics committee review board of all the participating hospitals, and in accordance with the Declaration of Helsinki. This trial was registered with ClinicalTrials.gov, number NCT02936089.

The present study included 207 newly diagnosed AE-AML patients from five medical centers in China from October 2016 to December 2021, with a median age of 33(14–67) years, and a ratio of male to female of 123:84. The detail characteristics were shown in Table 1. According to the mutation status of KIT and ASXL1, there were 105 patients in KIT−ASXL1− group, 87 in KIT+/ASXL1+ group, and 15 in KIT+ASXL1+ group. Among the three groups, significantly higher incidence of extramedullary infiltration (EMI) and BCOR mutation, a trend of higher bone marrow blasts and TP53 mutation, and lower median peripheral platelet count were found in KIT+ASXL1+ group, followed by KIT+/ASXL1+ group, compared to KIT−ASXL1− group. The details are shown in Table 1. These suggested co-mutation of KIT and ASXL1 might be associated with higher invasive and proliferative disease, further supporting the synergism of KIT and ASXL1 mutations in the development of AE-AML [19, 20].

**Table 1:** Clinical characteristics of the patients with AE-AML based on KIT/ASXL1 mutations.

	Total (*n* = 207)	KIT−ASXL1− (*n* = 105)	KIT+/ASXL1+ (*n* = 87)	KIT+ASXL1+ (*n* = 15)	*P*-value
Sex, Male/female	123/84	60/45	54/33	9/6	1.000
Median age (year, range)	33 (14–67)	34 (14–67)	32 (14–67)	36 (16–65)	0.697
*Peripheral blood cell count, median (range)*					
WBC (×10^9^/L)	12.4 (0.3–72.4)	12.0 (1.0–70.8)	12.4 (0.3–72.4)	18.6 (1.4–33.3)	0.549
HGB (g/L)	71 (26–148)	69.0 (37–148)	75 (26–127)	67 (38–88)	0.190
PLT (×10^9^/L)	25 (2–168)	27 (2–168)	24 (5–137)	16 (6–48)	0.057
BM blasts, median (range)	0.36 (0.01–0.94)	0.326 (0.01–0.94)	0.405 (0.08–0.91)	0.48 (0.03–0.94)	0.069
EMI (rate, %)	40 (19.3)	12 (11.4)	23 (27.1)	5 (38.5)	0.011
CD19+ (rate, %)	121 (58.5)	63 (60.0)	50 (57.6)	8 (46.2)	0.861
CD56+ (rate, %)	144 (69.6)	67 (63.8)	64 (74.1)	13 (84.6)	0.102
Karyotype	173	88	73	12	
ACAs (rate, %)	88 (50.9)	43 (48.3)	40 (55.7)	5 (40)	0.640
Loss of sex chromosomes (rate, %)	66 (38.2)	33 (37.1)	28 (38.6)	5 (40)	1.000
*Additional molecular mutations (%)*					
TET2	42 (20.3)	17 (16.2)	20 (23.0)	6 (40.0)	0.083
FLT3-ITD	21 (10.1)	12 (11.4)	8 (9.2)	1 (6.6)	0.773
EZH2	17 (8.2)	6 (5.7)	11 (12.6)	0	0.067
TET1	15 (7.2)	5 (4.8)	10 (11.5)	0	0.071
NRAS	8 (3.9)	4 (3.8)	4 (4.6)	0	0.736
KRAS	8 (3.9)	2 (1.9)	4 (4.6)	2 (13.3)	0.147
TP53	8 (3.9)	1 (1.0)	6 (6.9)	1 (6.6)	0.052
EVI1	8 (3.9)	2 (1.9)	6 (6.9)	0	0.129
CEBPA	8 (3.9)	5 (4.8)	2 (2.3)	1 (6.6)	0.810
KDM6A	8 (3.9)	5 (4.8)	3 (3.4)	0	0.590
RUNX1	7 (3.4)	1 (1.0)	6 (6.9)	0	0.053
DNMT3A	7 (3.4)	1 (1.0)	5 (5.7)	1 (6.6)	0.144
BCOR	5 (2.4)	0	4 (4.6)	1 (6.6)	0.036
ETV6	3 (1.4)	2 (1.9)	1 (1.1)	0	1.000

*WBC* white blood cell, *HGB* hemoglobin, *PLT* platelet, *BM* bone marrow, *EMI* extramedullary infiltration, *ACAs* additional chromosome abnormalities.

198 patients received 1–2 cycles of induction therapy [16, 22] and 188(95.0%) achieved CR/CRi. Finally, 195 patients acquired CR/CRi and went into risk-directed treatment (66 in the LR, 57 in the IR, and 72 in the HR), of whom 105 followed the design and 90 biased. Among those biased, 33 patients in the LR group received allo-SCT, 28 in the IR group and 29 in the HR group continued to receive CT. The detail is shown in Fig. 1. The cutoff date for the follow-up was December 31, 2022. With a median follow-up of 30(2–74) months, cumulatively 57 patients relapsed, with a cumulative incidence of relapse (CIR) of 28.8%. 49(24.7%) cases died, of whom 30 died of leukemia progression, 19 treatment-related mortality (TRM), including 10 died of transplantation-related complications. The rate of 3-year-overall survival (OS) was 63.8%, -progression-free survival (PFS) 57.2%, -CIR 32.4% (Table 2).

**Fig. 1:**
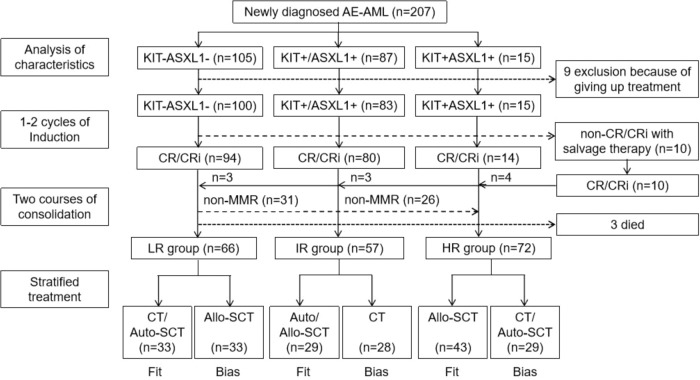
Schematic diagram of the study and treatment flow chart.

**Table 2:** Effect of KIT/ASXL1 mutations on the prognosis of the patients with AE-AML.

	Total (*n* = 198)	KIT-ASXL1−(*n* = 100)	KIT+/ASXL1+(*n* = 83)	KIT+ASXL1+(*n* = 15)	*P*-value
*Induction*					
CR/CRi after course 1	159 (80.3%)	85 (85.0%)	65 (78.3%)	9 (60.0%)	0.080
CR/CRi after course 2	188 (95.0%)	94 (94.0%)	80 (96.4%)	14 (93.3%)	0.597
MMR after course 2	31/121 (25.6%)	16/65 (24.6%)	12/50 (24.0%)	3/6 (50.0%)	0.390
*Consolidation*					
Relapse after course 2	15 (7.6%)	4 (4.0%)	7 (8.4%)	4 (26.7%)	0.018
CIR with follow-up	57 (28.8%)	25 (25%)	24 (28.9%)	8 (53.3%)	0.079
Death with follow-up	49 (24.7%)	17 (17.0%)	24 (28.9%)	8 (53.3%)	0.005
3 year-CIR	32.4%	31.7%	33.9%	70.4%	0.001
3 year-OS	63.8%	83.1%	67.4%	23.0%	0.000
3 year-PFS	57.2%	63.6%	59.9%	29.6%	0.001

*CR* complete remission, *CRi* CR without incomplete hematologic recovery, *MMR* main molecular remission, *CIR* cumulative incidence of relapse, *OS* overall survival, *PFS* progression-free survival.

First, we assessed the effect of KIT and ASXL1 mutations on the outcomes of AE-AML patients. Among the three groups based on KIT/ASXL1 mutations, there was no significant difference in the early response, including the CR/CRi rate after the first and second course of induction, and the MMR after the second cycle of induction (*P* > 0.05). Nevertheless, not only the early relapse during the first two courses of consolidation (KIT-ASXL1−4.0% vs. KIT+/ASXL1+8.4% vs. KIT+/ASXL1+26.7%, *P* = 0.018) but also the 3-year-CIR (31.7% vs. 33.9% vs. 70.4%, *P* = 0.001), in line with the result of Gray’s test (Fig. 2C), were significantly different. In addition, the mortality rate (17.0% vs. 28.9% vs. 53.3%, *P* = 0.005), 3-year-OS (83.1% vs. 67.4% vs. 23.0%, *P* < 0.001), and −PFS (63.6% vs. 59.9% vs. 29.6%, *P* = 0.001) were also significantly different (Table 2, Fig. 2A, B). These demonstrated that, in agreement with our previous report [2], KIT and ASXL1 mutations might have significantly adverse effects on the prognosis of AE-AML, and even worse in co-mutation patterns.

**Fig. 2:**
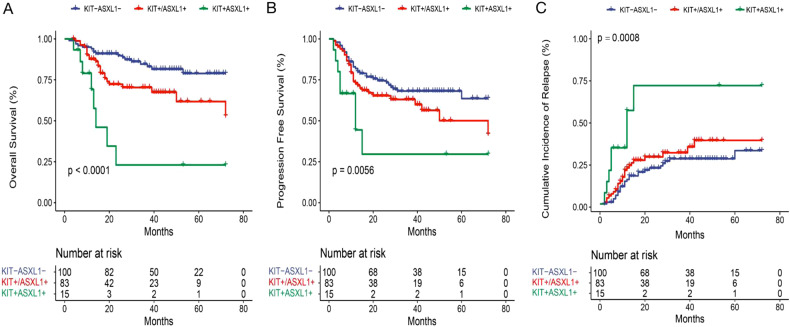
The impact of KIT/ASXL1 mutations on the outcomes of AE-AML patients. Overall survival (**A**), progression-free survival (**B**), and cumulative incidence of relapse (**C**) of the AE-AML patients based on KIT/ASXL1 mutations.

Next, we assessed the benefit of the risk-directed therapy based on KIT/ASXL1 mutations and MRD levels in AE-AML patients. Among the three risk groups, there was a significant difference in the incidence of recurrence (LR 16.7% vs. IR 26.3% vs. HR 43.1%, *P* < 0.001) and mortality (7.6% vs. 24.6% vs. 37.5%, *P* < 0.001) (Table 3). In accordance, the 3-year-CIR (22.9% vs. 31.9% vs. 48.5%, *P* < 0.001), -OS (90.5% vs. 71.4% vs. 57.8%, *P* < 0.001) and -PFS (74.4% vs. 63.9% vs. 42.3%, *P* < 0.001) were also significantly different (Table 3 and Fig. 3). When consolidation regimens were taken into consideration, the significant difference in the rate of OS, PFS and CIR among the three risk groups still existed in those with CT/auto-HSCT (Fig. 4A–C), but disappeared in the populations with allo-HSCT (Fig. 4D–F). It indicated that risk stratification based on KIT/ASXL1 mutations and MRD might significantly predict the relapse and survival of AE-AML patients, and be used to guide risk stratification therapy. Allo-HSCT could improve the outcomes of HR patients.

**Table 3:** The prognosis of the three risk groups based on KIT/ASXL1 mutations and MRD levels in AE-AML.

	LR group (*n* = 66)	IR group (*n* = 57)	HR group (*n* = 72)	*P*-value
Relapse	11 (16.7%)	15 (26.3%)	31 (43.1%)	0.000
Death	5 (7.6%)	14 (24.6%)	27 (37.5%)	0.000
3-year-CIR	22.9%	31.9%	48.5%	0.000
3-year-OS	90.5%	71.4%	57.8%	0.000
3-year-PFS	74.4%	63.9%	42.3%	0.000

*LR* low-risk, *IR* intermediate-risk, *HR* high-risk, *CIR* cumulative incidence of relapse, *OS* overall survival, *PFS* progression-free survival.

**Fig. 3:**
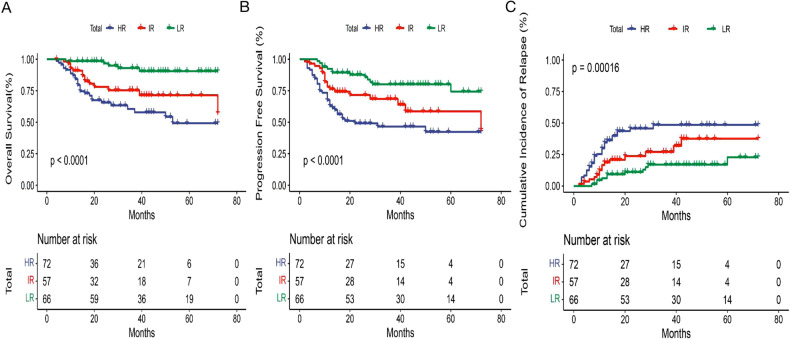
The impact of risk stratification based on KIT/ASXL1 mutations and MRD on the outcomes of AE-AML patients. Overall survival (**A**), progression-free survival (**B**), and cumulative incidence of relapse (**C**) of the AE-AML patients with different risk stratification based on KIT/ASXL1 mutations and MRD levels.

**Fig. 4:**
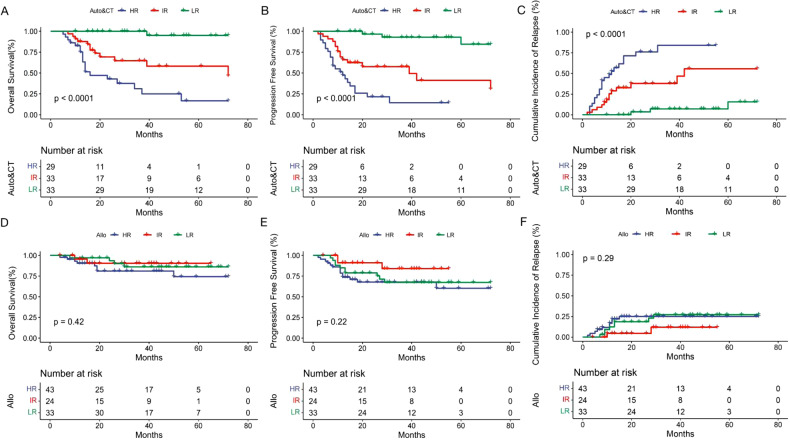
The impact of risk stratification on the outcomes of AE-AML patients with different consolidation. Overall survival (**A**, **D**), progression-free survival (**B**, **E**), and cumulative incidence of relapse (**C**, **F**) of the AE-AML patients with different risk stratification and different consolidation. **A**–**C** The patients with CT/auto-HSCT. **D**–**F** The patients with allo-HSCT.

As shown in Table 4 and Fig. 5, the patients having received the design treatment had apparently lower rate of relapse and death than those with treatment bias, especially significantly in the IR (3-year-CIR, 15.0% vs. 48.7%, *P* = 0.006; 3 year-OS, 92.3% vs. 52.5%, *P* = 0.006; 3-year-PFS, 81.8% vs. 46.8%, *P* = 0.005) and HR groups (3-year CIR, 25.0% vs. 84.1%, *P* < 0.001; 3-year OS, 74.3% vs. 25.0%, *P* < 0.001; 3-year PFS, 60.2% vs. 14.3%, *P* < 0.001). Even in the LR group, the 3-year-CIR (fit, 15.6% vs. bias, 27.2%, *P* = 0.048) and -PFS (fit, 84.4% vs. bias, 67.3%, *P* = 0.016) also significantly benefited from the stratification therapy. Furthermore, we subgrouped and analyzed the outcomes in the population with the design treatment and treatment bias, and found that the difference in the CIR, OS, and PFS among the three risk groups was narrowed in the patients who received the design treatment, while the difference was enlarged in the population with treatment bias (Fig. 6). Taking together, these indicated that risk-directed therapy benefited patients with significantly lower CIR and better OS and PFS than those without, also narrowed the gap of relapse and survival among the different risk groups.

**Table 4:** The impact of stratification therapy on the outcomes of the AE-AML patients based on KIT/ASXL1 mutations and MRD levels.

	LR group			IR group			HR group		
	Fit *n* = 33	Bias *n* = 33	*P*	Fit *n* = 29	Bias *n* = 28	*P*	Fit *n* = 43	Bias *n* = 29	*P*
Relapse	3 (9.1%)	8 (24.2%)	0.093	3 (0.3%)	12 (42.9%)	0.006	10 (23.3%)	21 (72.4%)	0.000
Death	1 (3.0%)	4 (12.1%)	0.178	2 (6.9%)	12 (42.9%)	0.002	8 (18.6%)	9 (31.0%)	0.000
3y-CIR	15.6%	27.2%	0.048	15.0%	48.7%	0.006	25.0%	84.1%	0.000
3y-OS	95.0%	86.1%	0.160	92.3%	52.5%	0.006	74.3%	25.0%	0.000
3y-PFS	84.4%	67.3%	0.016	81.8%	46.8%	0.005	60.2%	14.3%	0.000

*LR* low-risk, *IR* intermediate-risk, *HR* high-risk, *CIR* cumulative incidence of relapse, *OS* overall survival, *PFS* progression-free survival.

**Fig. 5:**
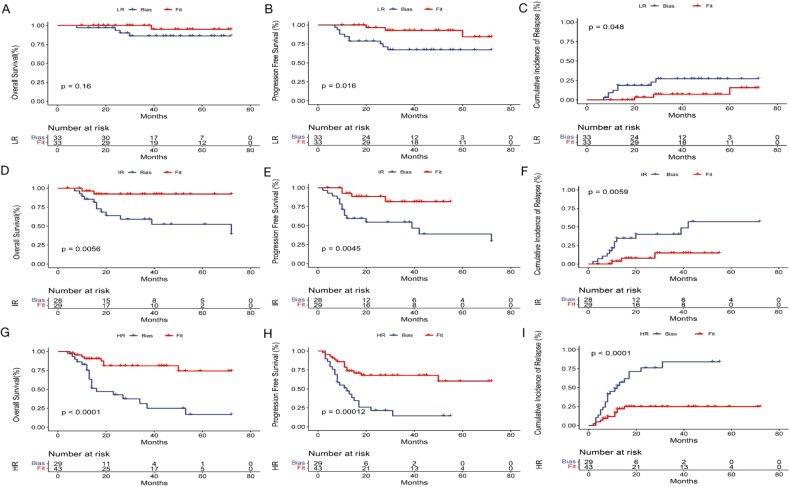
Stratification therapy improved the outcomes of AE-AML patients compared to treatment bias. Stratification therapy for the AE-AML patients based on KIT/ASXL1 mutations and MRD levels and comparison of overall survival, progression-free survival and cumulative incidence of relapse in the LR (**A**–**C**), IR (**D**–**F**), and HR (**G**–**I**) populations with treatment fit versus treatment bias.

**Fig. 6:**
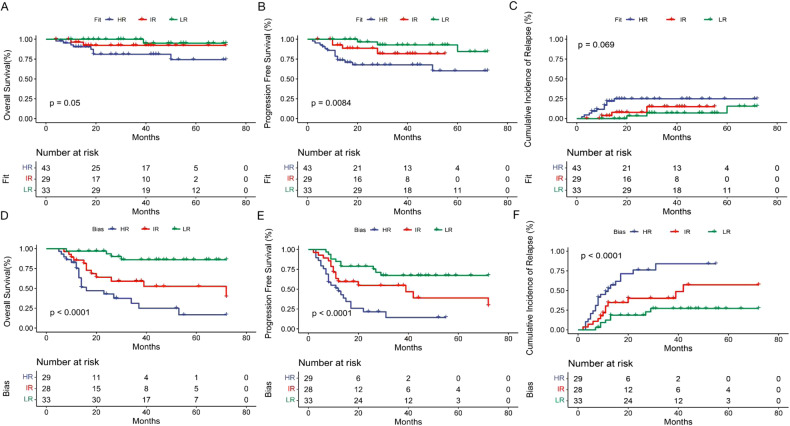
Stratification therapy narrowed the gap in the outcomes among the three risk groups. The difference in the overall survival (**A**, **D**), progression-free survival (**B**, **E**), and cumulative incidence of relapse (**C**, **F**) among the three risk groups was narrowed in the patients with stratification therapy according to the design (**A**–**C**), while was enlarged in the patients with treatment bias (**D**–**F**).

In conclusion, AE-AML patients might have higher invasive and proliferative characteristics and worse outcomes with increasing numbers of KIT/ASXL1 mutations. Risk stratification and stratification therapy of patients based on a combination of KIT and ASXL1 mutations with MRD might improve the prognosis of patients with AE-AML. More multi-center prospective studies are needed to confirm the current results.

## Data Availability

The data are available from the corresponding author. Please contact the corresponding author by e-mail: yugpp@163.com.
